# Comparison of Left Atrial Appendage Occlusion versus Non-Vitamin-K Antagonist Oral Anticoagulation in High-Risk Atrial Fibrillation: An Update

**DOI:** 10.3390/jcdd8060069

**Published:** 2021-06-11

**Authors:** Shaojie Chen, K. R. Julian Chun, Zhiyu Ling, Shaowen Liu, Lin Zhu, Jiazhi Wang, Alexandra Schratter, Willem-Jan Acou, Márcio Galindo Kiuchi, Yuehui Yin, Boris Schmidt

**Affiliations:** 1 Cardioangiologisches Centrum Bethanien (CCB), Kardiologie, Medizinische Klinik III, Agaplesion Markus Krankenhaus, Akademisches Lehrkrankenhaus der Goethe-Universität Frankfurt am Main, 60431 Frankfurt am Main, Germany; j.chun@ccb.de (K.R.J.C.); b.schmidt@ccb.de (B.S.); 2 Die Sektion Medizin, Universität zu Lübeck, 23538 Lübeck, Germany; 3 Department of Cardiology, The Second Affiliated Hospital of Chongqing Medical University, Chongqing 400000, China; lingzy1977@163.com (Z.L.); yinyh63@163.com (Y.Y.); 4 Department of Cardiology, Shanghai General Hospital, Shanghai Jiao Tong University School of Medicine, Shanghai 200000, China; shaowen.liu@hotmail.com; 5 Medizinisch-Geriatrische Klinik, Agaplesion Markus Krankenhaus, Akademisches Lehrkrankenhaus der Goethe-Universität Frankfurt am Main, 60431 Frankfurt am Main, Germany; l.qd.zhu@gmail.com; 6 Intensivmedizin, Charité—Universitätsmedizin Berlin, 10117 Berlin, Germany; wjz227@hotmail.com; 7 Medizinische Abteilung mit Kardiologie, Krankenhaus Hietzing Wien, 1130 Vienna, Austria; alexandra.schratter@gmx.at; 8 Department of Cardiology, AZ Delta, 8800 Roeselare, Belgium; wjacou@yahoo.com; 9 School of Medicine-Royal Perth Hospital Unit, University of Western Australia, Perth 6907, Australia; marciokiuchi@gmail.com

**Keywords:** atrial fibrillation, left atrial appendage occlusion, stroke, anticoagulation

## Abstract

Transcatheter left atrial appendage occlusion (LAAO) is non-inferior to vitamin K antagonists (VKAs) in preventing thromboembolic events in atrial fibrillation (AF). Non-vitamin K antagonists (NOACs) have an improved safety profile over VKAs; however, evidence regarding their effect on cardiovascular and neurological outcomes relative to LAAO is limited. Up-to-date randomized trials or propensity-score-matched data comparing LAAO vs. NOACs in high-risk patients with AF were pooled in our study. A total of 2849 AF patients (LAAO: 1368, NOACs: 1481, mean age: 75 ± 7.5 yrs, 63.5% male) were enrolled. The mean CHA2DS2-VASc score was 4.3 ± 1.7, and the mean HAS-BLED score was 3.4 ± 1.2. The baseline characteristics were comparable between the two groups. In the LAAO group, the success rate of device implantation was 98.8%. During a mean follow-up of 2 years, as compared with NOACs, LAAO was associated with a significant reduction of ISTH major bleeding (*p* = 0.0002). There were no significant differences in terms of ischemic stroke (*p* = 0.61), ischemic stroke/thromboembolism (*p* = 0.63), ISTH major and clinically relevant minor bleeding (*p* = 0.73), cardiovascular death (*p* = 0.63), and all-cause mortality (*p* = 0.71). There was a trend toward reduction of combined major cardiovascular and neurological endpoints in the LAAO group (OR: 0.84, 95% CI: 0.64–1.11, *p* = 0.12). In conclusion, for high-risk AF patients, LAAO is associated with a significant reduction of ISTH major bleeding without increased ischemic events, as compared to “contemporary NOACs”. The present data show the superior role of LAAO over NOACs among high-risk AF patients in terms of reduction of major bleeding; however, more randomized controlled trials are warranted.

## 1. Introduction

Atrial fibrillation (AF) is the most common cardiac arrhythmia worldwide. AF is a major risk factor for stroke/thromboembolism, cardiovascular morbidity, and mortality and contributes significantly to the healthcare burden. Nowadays, oral anticoagulation (OAC) with vitamin K antagonists (VKAs) or non-vitamin K antagonists (NOACs) is the standard of care to prevent stroke or thromboembolism in patients with AF [1].

However, a growing number of patients are intolerant or have contraindications for long-term OAC therapy. Percutaneous left atrial appendage occlusion (LAAO), as a catheter-based interventional strategy to prevent left atrial appendage (LAA) thrombus formation or migration, has emerged to treat such patient group. Pivotal large randomized trials have demonstrated that LAAO is non-inferior to VKA in terms of stroke prevention [2].

As compared with VKAs, NOACs have similar efficacy and improved safety profile; however, directly comparative data of NOACs versus LAAO on clinical outcomes are limited [1].

We conducted a systematic review and investigated a pooled analysis of existing comparative studies to assess the safety and efficacy of LAAO versus NOACs in preventing major cardiovascular and neurological adverse events in patients with AF.

## 2. Methods

The inclusion criteria were based on the patient, intervention, comparison, and outcome (PICO) principles as follows: (1) patients of interest: atrial fibrillation; (2) intervention and comparison: LAAO vs. NOACs; (3) outcome: major cardiovascular and neurological adverse events. To minimize bias, only randomized trials and propensity-score-matched cohort studies were included.

Data search was conducted using the PubMed and www.clinicaltrials.gov databases until February 2021, with the keywords: [“atrial fibrillation”] and [“left atrial appendage occlusion” OR “left atrial appendage closure”] and [“non-vitamin K oral anticoagulant” OR “direct oral anticoagulant” OR “novel oral anticoagulant”].

The study outcomes included (1) ischemic stroke and thromboembolism (ISTE), (2) major bleeding (based on the International Society on Thrombosis and Haemostasis (ISTH)), or clinically relevant bleeding, and (3) all-cause mortality.

### Data Collection and Quality Assessment

Data regarding baseline characteristics, treatment, follow-up, and outcomes were extracted. The quality assessment of the included data was in accordance with the recommendation of the Cochrane Handbook [3]. The key items for data quality assessment were (1) randomized trial, (2) double blinded, (3) clear definition of the study population, (4) clear definition of study comparison, (5) clear definition of outcomes assessment, (6) appropriate statistical method used, (7) no selective loss of data analysis, and (8) important confounders identified.

## 3. Statistical Analysis

The categorical variables were expressed as percentages and estimated by odds ratio (OR), and continuous variables were expressed as mean and standard deviation (SD). Considering the intrinsic variation and different sample sizes between individual studies, the ORs were estimated using a random effects model for all comparisons. Inter-study heterogeneity was quantified by the statistic value I^2^, estimated by the Q test. For non-RCTs, patients’ data after matching propensity scores were included. All *p* values were two-tailed, and the statistical significance was set at 0.05. The statistical analyses were conducted using the Revman (Review Manager, Version 5.4. The Nordic Cochrane Centre, the Cochrane Collaboration, Copenhagen, Denmark).

## 4. Results

### 4.1. Baseline Characteristics

As shown in Table 1, a total of 2849 AF patients (LAAO: 1368, NOACs: 1481) from three studies (one RCT, two propensity-score-matched studies) were included [4,5,6]. The baseline characteristics of the included studies are summarized in Table 1. Overall, the mean age was 75 ± 7.5 yrs, 63.5% were male, the mean CHA2DS2-VASc score was 4.3 ± 1.7, and the mean HAS-BLED score was 3.4 ± 1.2. Of the included patients, 32.2% had previous stroke/TIA, 17% had concurrent renal dysfunction. The baseline characteristics appeared comparable (Table 2). In the LAAO group, the device was successfully implanted in 98.8% of the patients.

### 4.2. Clinical Outcomes

**Figure 1:**
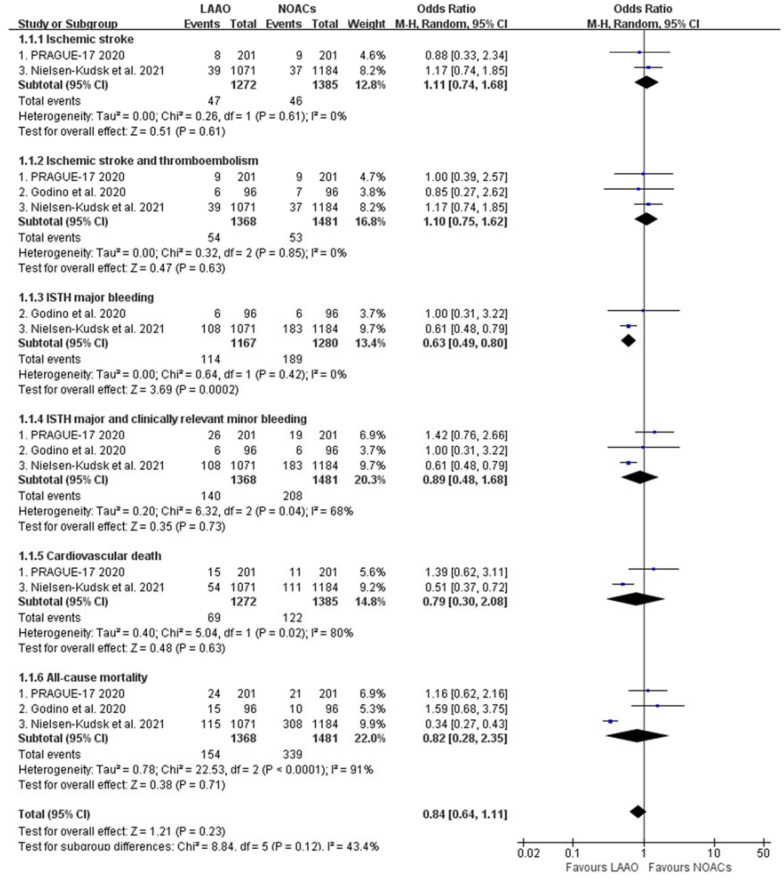
LAAO vs. NOACs: comparisons of major cardiovascular and neurological outcomes in high-risk AF.

The mean follow-up was 2 years. As compared with NOACs, LAAO was associated with a significant reduction of ISTH major bleeding (OR: 0.63, 95% CI: 0.49–0.80, *p* = 0.0002), with a relative risk reduction of 37% and absolute risk reduction of 5% (2.5%/yr) (Figure 1).

There were no significant differences in terms of ischemic stroke (OR: 1.11, 95% CI: 0.74–1.68, *p* = 0.61), ischemic stroke/thromboembolism (OR: 1.10, 95% CI: 0.75–1.62, *p* = 0.63), ISTH major and clinically relevant minor bleeding (OR: 0.89, 95%CI: 0.48–1.68, *p* = 0.73), cardiovascular death (OR: 0.79, 95% CI: 0.30–2.08, *p* = 0.63), and all-cause mortality (OR: 0.82, 95% CI: 0.28–2.35, *p* = 0.71). There was a trend toward reduction of combined major cardiovascular and neurological endpoints in the LAAO group (OR: 0.84, 95% CI: 0.64–1.11, *p* = 0.12) (Figure 1).

LAAO procedure or device related serious complications are summarized in Table 3. There were 1.1% pericardial effusion/tamponade, 0.37% thromboembolism, 0.22% device dislodgement, 0.66% puncture site complications requiring intervention, and 0.37% death.

## 5. Discussion

**Figure 2:**
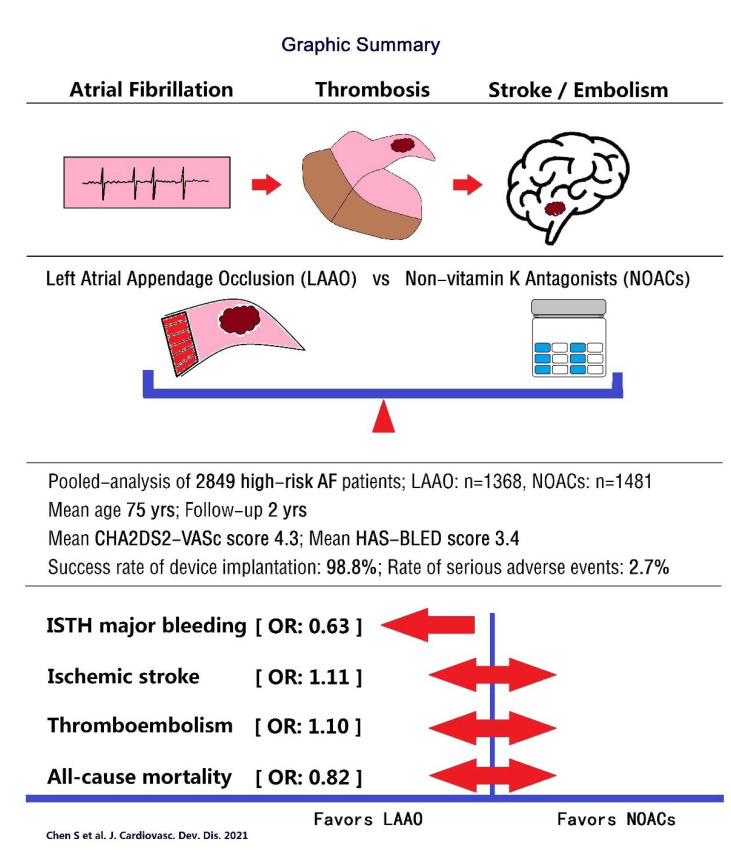
Graphic summary of the present study.

The main findings of the present analysis are shown in Figure 2 as a graphic summary.

Propensity-score matching is a statistical method to mimic randomization and estimate the effect of treatment by accounting for the covariates. Pooled analysis, which quantitatively combines single results into a summary estimate, is a foundational technique for evidence-based medicine. The known advantage of LAAO is a reduced bleeding risk by avoiding long-term anticoagulation while still providing continuous protection from ischemic stroke and thromboembolism, as compared to VKA [2]. With the continuous advancement of medical technology, new devices with improved designs facilitate the procedural workflow, and the implant success rate has been significantly increased based on cumulative experience [7,8,9,10,11,12]. Recent studies also suggested that LAAO may be considered in AF patients after electrical LAA isolation for rhythm control due to increased thromboembolic risk even under OAC [13,14,15].

Historically, Vitamin K antagonist (VKA) is the traditional anticoagulant and is effective for the prevention of ischemic stroke in patients with AF. However, the known limitations of VKAs include interactive effects with foods and drugs, unpredictable anticoagulant response, and requiring laboratory monitoring on a regular basis. These limitations cause problems for many patients and result in poor clinical compliance with anticoagulant therapy.

Non-vitamin-K antagonist oral anticoagulation or novel oral anticoagulant drugs (NOACs) including direct thrombin inhibitors and factor Xa inhibitors have been therefore developed. The NOACs have the potential to overcome the limitations of VKAs, i.e., shorter half-life, more controllable administration, fewer food and drug interactions, more predictable anticoagulant effects, and without the need for laboratory monitoring. The landmark trials of NOACs, i.e., RE-LY trial (Dabigatran), ARISTOTLE trial (Apixaban), ROCKET-AF trial (Rivaroxaban), and ENGAGE AF-TIMI 48 trial (Edoxaban), have demonstrated that NOACs are at least non-inferior to warfarin with respect to the prevention of ischemic stroke or systemic embolism and may be associated with significantly lower rates of bleeding and cardiovascular death [16,17,18,19,20].

As a catheter-based interventional strategy, LAAO serves as an alternative approach to prevent thrombus formation in the LAA for those AF patients who are intolerant or contraindicative for long-term OAC therapy. Large randomized trials have demonstrated that LAAO provides stroke prevention in AF comparable to warfarin, with additional reductions in major bleeding [2]. As mentioned above, NOACs have also similar efficacy and lower bleeding risk as relative to VKAs. Thus, a direct comparison between NOACs vs. LAAO appears clinically relevant.

The present large pooled analysis demonstrated that LAAO was associated with a substantially lower risk of major bleeding, as compared with NOACs. The results were derived from a patient group with a mean age of 75 yrs and a mean CHA2DS2-VASc score/HAS-BLED score of 4.3 ± 1.7/3.4 ± 1.2. About one-third of the patients had previous stroke/TIA, and one-fifth of the patients had concurrent renal dysfunction. These baseline characteristics represented a selected population who carried a high risk of all major cardiovascular events. In the PRAGUE-17 Trial [4] and in the Amulet observational study [6], the Kaplan–Meier analysis for clinically relevant bleeding or major bleeding began to show a significant difference at 1 year, when the majority of the patients in the LAAO arm were under single antiplatelet therapy instead of NOACs or dual antiplatelet therapy.

There was a trend toward reduction of combined major cardiovascular and neurological endpoints in the LAAO, relative to the NOACs group, which was mainly driven by the lower major bleeding events. With respect to other relevant outcomes, such as ischemic stroke, thromboembolism, bleeds, cardiovascular or all-cause mortality, further investigations with a large sample and long-term follow-up are warranted, e.g., OPTION (NCT03795298), OCCLUSION-AF (NCT03642509), CLOSURE-AF (NCT03463317), CATALYST (NCT04226547), and CHAMPION-AF (NCT04394546) trials. Table 4 summarizes these ongoing registered clinical trials.

Notably, our pooled analysis showed 98.8% of successful LAAO device implantation and 2.7% of major procedure-related complications. As compared with earlier randomized trials, such contemporary procedural data demonstrated that a higher implant success rate and lower risk of complications may reflect the improvement in patient selection and operator/center experience.

Is LAAO cost-effective? A previous study investigated the cost-effectiveness of LAAO, compared with VKAs or NOACs, for the prevention of stroke in AF patients. In this study, a Markov model was constructed using data from the pivotal NOACs trials and the LAAO trials. Costs were based on 2016 US Medicare reimbursement rates and the literature. The analysis of cost-effectiveness was conducted over a lifetime (20 years) horizon. The study demonstrated that, initially, the procedure costs make LAAO higher cost than VKAs and NOACs; however, in the long-term perspective (within 10 years), LAAO offers more quality-adjusted life years and has lower total costs, making LAAO the cost-effective treatment strategy for prevention of stroke in AF [21]. Similarly, a multicenter analysis from Canada demonstrated that LAAO, as a stroke preventative therapy for AF, is a cost-effective alternative to aspirin in patients with contraindications to OAC in a long-term perspective [22].

Nevertheless, the decision making of LAAO should be individualized and be based on full consideration of the benefit and risk. With current evidence, it should not extrapolate that LAAO can be generalized to all AF patients and replace NOACs in the full population. However, the present data at least support the use of LAAO in high-risk AF patients who are inappropriate for long-term NOACs.

## 6. Conclusions

In high-risk AF patients, LAAO appears to be safe and is associated with a significant reduction of ISTH major bleeding without increased ischemic events, as compared to “contemporary NOACs.” The present data show the superior role of LAAO over NOACs among high-risk AF patients in terms of reduction of major bleeding; however, more randomized controlled trials are warranted.

## Figures and Tables

**Table 1:** Baseline characteristics in included studies.

	PRAGUE-17 Trial 2020		Godino et al., 2020		Nielsen-Kudsk et al., 2021	
Design	Randomized Trial		Propensity-Score Matching		Propensity-Score Matching	
Intervention	LAAO	NOACs	LAAO	NOACs	LAAO	NOACs
Sample size	201	201	96	96	1071	1184
Age, yrs	73.4 ± 6.7	73.2 ± 7.2	73.8 ± 7.1	75.3 ± 6.8	75.1 ± 8.5	75.1 ± 10.5
Male, n (%)	134 (66.7)	130 (64.7)	54 (56.2)	78 (81.3)	687 (64.2)	727 (61.4)
Weight, kg	86.9 ± 17.6	88.1 ± 16.2	/	/	/	/
BMI, kg/m^2^	/	/	25.7 ± 3.6	26.4 ± 4.3	/	/
Indication for treatment	AF	AF	AF	AF	AF	AF
CHA_2_DS_2_-VASc	4.7 ± 1.5	4.7 ± 1.5	4.3 ± 1.5	4.3 ± 1.5	4.2 ± 1.6	4.3 ± 1.7
HAS-BLED	3.1 ± 0.9	3.0 ± 0.9	3.5 ± 0.7	3.5 ± 0.6	3.3 ±1.0	3.4 ± 1.2
Heart failure	88 (43.8)	90 (44.8)	/	/	178 (16.6)	223 (18.9)
LVEF, %	53.3 ± 12.6	52.9 ± 12.1	51.3 ± 10.8	52.1 ± 11.7	/	/
Hypertension	186 (92.5)	186 (92.5)	80 (83.3)	90 (93.8)	896 (83.7)	1023 (86.5)
Diabetes mellitus	73 (36.3)	90 (44.8)	24 (25)	23 (24)	333 (31.1)	424 (35.8)
History of ischemic Stroke/TIA	66 (32.8)	63 (31.3)	41 (43.2)	37 (38.5)	333 (31.1)	376 (31.8)
Coronary artery disease	/	/	/	/	346 (32.3)	402 (33.9)
History of MI	30 (14.9)	39 (19.4)	11 (11.5)	23 (24.5)	/	/
Renal dysfunction	/	/	36 (46.8)	34 (35.4)	149(13.9)	169 (14.3)
Liver dysfunction	/	/	4 (4.2)	4 (4.2)	51 (4.8)	77 (6.5)
Renal or liver dysfunction	47 (23.4%)	44 (21.9%)	/	/	/	/
Devices/NOACs	Amulet (61.3%) Watchman (35.9%) Watchman-FLX (2.8%)	Dabigatran (4%) Apixaban (95.5%) Rivaroxaban (0.5%)	Watchman (33.7%) AMPLATZER (22.3%) Amulet (44%)	Dabigatran (41%) Apixaban (41%) Rivaroxaban (18%).	Amulet (100%)	NOACs
Procedural LAAO leak >5mm	4(2.2%)		0		0.7% (>3 mm)	
Success rate of LAAO	96.8%		100%		99.1%	

**Table 2:** Pooled baseline characteristics.

	LAAO	NOACs
Sample Size	1368	1481
Age, yrs	74.8 ± 8.2	74.9 ± 9.9
Male, n (%)	875 (64%)	935 (63%)
Weight, kg	86.9 ± 17.6 (n = 201)	88.1 ± 16.2 (n = 201)
BMI, kg/m^2^	25.7 ± 3.6 (n = 96)	26.4 ± 4.3 (n = 96)
CHA_2_DS_2_-VASc	4.3 ± 1.6	4.4 ± 1.7
HAS-BLED	3.3 ± 1	3.3 ±1.1
Heart Failure	266 (20.9%) (n = 1272)	313 (22.6%) (n = 1385)
LVEF, %	52.7 ± 12.1 (n = 297)	52.6 ± 12 (n = 297)
Hypertension	1162 (84.9%)	1299 (87.7%)
Diabetes Mellitus	430 (31.4%)	537 (36.3%)
History of Ischemic Stroke/TIA	440 (32.2%)	476 (32.1%)
Ischemic Heart Disease	387 (28.3%)	464 (31.3%)
Renal Dysfunction	185 (15.9%) (n = 1167)	203 (15.9%) (n = 1280)
Liver Dysfunction	55 (4.7%) (n = 1167)	81 (6.3%) (n = 1280)

**Table 3:** LAAO implant success and LAAO procedure- or device-related serious complications.

	Pooled
Sample Size	1368
Implant Success Rate	98.8%
Pericardial Effusion/Tamponade	15 (1.1%)
Thromboembolism	5 (0.37%)
Device Dislodgement	3 (0.22%)
Puncture Site Complications Requiring Intervention	9 (0.66%)
Death	5 (0.37%)
Reasons of Death	2 from tamponade 2 from myocardial infarction 1 from cardiorespiratory arrest

**Table 4:** Ongoing registered clinical trials (refer to: https://clinicaltrials.gov).

Trials	Trial Number	Design	Comparison	Sample Size	Planned Follow-Up
OPTION	(NCT03795298)	Randomized	WATCHMAN FLX LAAO vs. OACs	1600	36 months
OCCLUSION-AF	(NCT03642509)	Randomized	Amulet or Watchman LAAO vs. NOACs	750	5 years
CLOSURE-AF	(NCT03463317)	Randomized	LAAO devices vs. OACs	1512	24 months
CATALYST	(NCT04226547)	Randomized	Amulet LAAO vs. NOACs	2650	2 years
CHAMPION-AF	(NCT04394546)	Randomized	WATCHMAN FLX LAAO vs. NOACs	3000	36 months

Abbreviations list for these clinical trials: (1) Comparison of anticoagulation with left atrial appendage closure after af ablation (OPTION); (2) Left atrial appendage occlusion versus novel oral anticoagulation for stroke prevention in atrial fibrillation (Occlusion-AF); (3) Left atrial appendage closure in patients with atrial fibrillation compared to medical therapy (CLOSURE-AF); (4) Amplatzer Amulet LAAO vs. NOAC (CATALYST); (5) Left atrial appendage closure vs. non-vitamin K oral anticoagulants (CHAMPION-AF).

## Data Availability

This was a meta-analysis of published data which are available from the included studies.

## Abbreviations

LAAO: left atrial appendage occlusion; NOACs: non-vitamin-k antagonist oral anticoagulation; BMI: body mass index; LVEF: left ventricular ejection fraction; MI: myocardial infarction.
